# CALL Score and RAS Score as Predictive Models for Coronavirus Disease 2019

**DOI:** 10.7759/cureus.11368

**Published:** 2020-11-07

**Authors:** Sultan M Kamran, Zill-e-Humayun Mirza, Hussain Abdul Moeed, Arshad Naseem, Maryam Hussain, Imran Fazal, Farrukh Saeed, Wasim Alamgir, Salman Saleem, Sidra Riaz

**Affiliations:** 1 Pulmonology, Pak Emirates Military Hospital, Rawalpindi, PAK; 2 Internal Medicine, Pak Emirates Military Hospital, Rawalpindi, PAK; 3 Critical Care, Pak Emirates Military Hospital, Rawalpindi, PAK; 4 Medicine, Pak Emirates Military Hospital, Rawalpindi, PAK; 5 Gastroenterology, Pak Emirates Military Hospital, Rawalpindi, PAK; 6 Neurology, Pak Emirates Military Hospital, Rawalpindi, PAK; 7 Infectious Disease, Pak Emirates Military Hospital, Rawalpindi, PAK

**Keywords:** covid-19, prediction model, call score, prognostic markers, respiratory rate, oxygen saturation, a-a gradient, minimal exercise desaturation, comorbidities

## Abstract

Background: Coronavirus disease 2019 (COVID-19) is a novel infectious disease of multi-system involvement with significant pulmonary manifestations. So far, many prognostic models have been introduced to guide treatment and resource management. However, data on the impact of measurable respiratory parameters associated with the disease are scarce.

Objective: To demonstrate the role of Comorbidity-Age-Lymphocyte count-Lactate dehydrogenase (CALL) score and to introduce Respiratory Assessment Scoring (RAS) model in predicting disease progression and mortality in COVID-19.

Methodology: Data of 252 confirmed COVID-19 patients were collected at Pak Emirates Military Hospital (PEMH) from 10th April 2020 to 31st August 2020. The CALL score and proposed factors of RAS model, namely respiratory rate, oxygen saturation at rest, alveolar arterial gradient and minimal exercise desaturation test, were calculated on the day of admission. Progression of disease was defined and correlated with measured variables. Univariate and multivariate Cox regression analysis for each variable, its hazard ratio (HR) and 95% confidence interval (CI) were calculated, and a nomogram was made using the high-risk respiratory parameters to establish the RAS model.

Results: Progression of disease and death was observed in 124 (49.2%) and 49 (19.4%) patients, respectively. Presence of more than 50% of chest infiltrates was significantly associated with worsening disease and death (p-value <0.001). Death was observed in 100% of patients who had critical disease category on presentation. Regression analysis showed that the presence of comorbidity (n: 180), in contrast to other variables of CALL score, was not a good prognosticator of disease severity (p-value: 0.565). Nonetheless, the CALL model itself was validated to be a reliable prognostic indicator of disease progression and mortality. Some 10 feet oxygen desaturation test (HR: 0.99, 95%CI: 0.95-1.04, p­-value: 0.706) was not a powerful predictor of the progression of disease. However, respiratory rate of more than 30 breaths/minute (b/m) (HR: 3.03, 95%CI: 1.77-5.19), resting oxygen saturation of less than 90% (HR: 2.41, 95%CI: 1.15-5.06), and an elevated alveolar-arterial oxygen gradient (HR: 2.14, 95%CI: 1.04-4.39) were considered statistically significant high-risk predictors of disease progression and death, in the formed RAS model. The model resulted in 85% (95%CI: 80%-89%) of area under the receiver operating characteristic curve (AUROC), with substantial positive (76%, 95%CI: 68%-83%) and negative predictive values (80%, 95%CI: 73%-87%) for a cutoff value of seven. Patients with higher CALL and RAS scores also resulted in higher mortality.

Conclusion: CALL and RAS scores were strongly associated with progression and mortality in patients with COVID-19.

## Introduction

The novel coronavirus, severe acute respiratory syndrome coronavirus 2 (SARS-CoV-2), first described in December 2019 has spread to over 210 countries. The World Health Organization (WHO) announced the coronavirus disease 2019 (COVID-19) a pandemic on 11th March 2020 and up till now over 40 million substantiated cases of this illness have been recorded, with more than one million deaths [1]. COVID-19 is a virulent multi-system disease manifesting primarily as a respiratory illness with symptoms ranging from fever, cough, fatigue, sore throat and shortness of breath, to impending critical disease requiring mechanical ventilation, or ending in death [2]. People with underlying conditions such as hypertension (HTN), cardiovascular disease, diabetes mellitus (DM), chronic obstructive pulmonary disease (COPD), and malignancy have an increased probability of advancement to severity and greater mortality [3]. Considering high fatality (61.5%) in critically ill patients with SARS-CoV-2 infection [4], it is a matter of utmost concern to identify mild and moderate cases earlier and predict disease progression based on risk factors so that an early escalation of treatment is ensured.

Various predictive models for COVID-19 have been designed during this pandemic, which are now accessible in academic literature to aid medical decision making [5]. A predictive model, Comorbidity-Age-Lymphocyte count-Lactate dehydrogenase (CALL), has been devised to anticipate disease progression [6]. However, the CALL score does not cater for respiratory assessment parameters, which are also paramount in predicting disease progression and mortality [7]. It has been observed that around 15% of cases of COVID-19 are complicated by interstitial pneumonia and a variable degree of respiratory failure [2].

Early disclosure of increasing respiratory involvement of COVID-19 can help physicians to enact necessary measures including treatment escalation. For this purpose, an alternative model called the Respiratory Assessment Scoring (RAS) was devised in this study. Various easy-to-calculate clinical and laboratory respiratory parameters, such as respiratory rate (RR), resting oxygen (O2) saturation, alveolar-arterial oxygen gradient (A-a gradient), and minimal exercise desaturation test were employed in order to generate potential high-risk factors to formulate the scoring model.

## Materials and methods

Study design and setting

This single-center cross-sectional study was carried out at the department of pulmonology of the Pak Emirates Military Hospital (PEMH), Rawalpindi, Pakistan from 1st April 2020 to 31st August 2020 after approval by the institutional ethical review committee (ERC). Data of 252 hospitalized patients afflicted with COVID-19 admitted in the pulmonology ward were included. Informed consent was given by all subjects.

Inclusion and exclusion criteria

Inclusion criteria included: (1) COVID-19 diagnosed by real time-polymerase chain reaction (RT-PCR) positivity for SARS-CoV-2, (2) mild disease with comorbidities, (3) moderate, severe, and critical illness, (4) 10-80 years of age, and (5) both genders. Exclusion criteria comprised: (1) asymptomatic or mild disease with no comorbidity, (2) cases who expired within 24 hours of hospitalization, (3) advanced malignancies with life expectancy less than six months, and (4) presence of co-infections.

Study definitions

On admission, patients were stratified into disease categories based on criteria set by WHO [8]. Mild disease meant patients with uncomplicated upper respiratory tract viral infection having nonspecific manifestations such as low-grade fever, cough, body aches, muscle pain, sore throat, nasal congestion, headache, diarrhea, nausea, vomiting, anosmia, and ageusia. Moderate disease was defined as a case with lung infiltrates less than 50% of the total lung fields on X-ray chest and peripheral ground-glass opacities (GGOs) on high-resolution computed tomography (HRCT) chest, and no evidence of hypoxemia or severe pneumonia. Severe disease was operationally described as COVID-19 pneumonia with evidence of hypoxemia (RR more than 30 breaths/minute (b/m) or partial pressure of oxygen (PaO2) less than 80 mmHg on arterial blood gases (ABGs) or lung infiltrates being over 50% of the lung fields). Critical disease was determined if there was evidence of either respiratory failure (PaO2 <60 mmHg) or multiorgan dysfunction syndrome (MODS) measured by the sequential organ failure assessment (SOFA) score of over 10 or septic shock (systolic blood pressure less than 90 mmHg or less than 40 mmHg of baseline in hypertensive or urine output less than 0.5 mL/kg/hour). Comorbidity was described as any chronic health issue for which the patient was using medications previously. Progression of the disease meant an increasing demand for oxygen and/or ascending up to higher disease severity category or death because of COVID-19 related complications.

Data structure and collection

As per institutional approach, every subject with comorbidities or moderate, severe and critical illness at the time of admission and during hospitalization underwent monitoring and evaluation. This process included, but was not restricted to, vital signs monitoring (including pulse oximetry), baseline laboratory testing (complete blood count, coagulation profile, liver and renal function tests), blood testing for cytokine release storm [ferritin, D-dimers, lactate dehydrogenase (LDH), interleukin-6 (IL-6), absolute lymphocyte count (ALC)], tests for cardiac injury biomarkers [troponins, creatine kinase-MB (CKMB)], HRCT chest, and ABGs analysis. Respiratory specimens were also investigated for co-infections (influenza, avian influenza, respiratory syncytial virus, adenovirus, parainfluenza) via PCR analysis. During the stay in hospital, many of the above parameters were repeated when deemed necessary. A 10 feet walk O2 desaturation test was devised for minimal exercise desaturation assessment considering patient safety and disease condition since many already known exercise tests were deemed excessive for admitted patients [9]. The 10 feet walk O2 desaturation test was carried out under supervised care and only after the patient’s consent. An oxygen desaturation of equal to and greater than three percent was notable for this test.

Extracted data of all the subjects included in the study comprised of variables such as age, gender, comorbidities, contact history, types of symptoms, HRCT chest pattern, percentage of lung involvement, and initial disease category. For estimation of the CALL score added information, including ALC and LDH levels at the day of admission (day zero) were obtained. For respiratory assessment, variables such as RR, O2 saturation with pulse oximetry at rest and supported by ABGs, A-a gradient and 10 feet walk O2 desaturation test results were retrieved for day zero. To note the progression of illness, variables such as the final disease category and mortality were noted.

Statistical analysis

Data were analyzed using R software version 4.0.2 (R Foundation for Statistical Computing, Vienna, Austria). Continuous variables were checked for skewness with the Shapiro-Wilk test [10] and were expressed as medians [interquartile range (IQR)] and compared using the Mann-Whitney test. Categorical variables were presented as numbers (percentage) and correlated by the chi-square test. All statistical results were deemed significant at a p-value <0.05. Univariate and multivariate Cox regression analysis was used for assessing the relevance of each variable of the CALL score and proposed respiratory parameters of the RAS model to verify and investigate, respectively, the high-risk factors for progression of illness with their hazard ratio (HR) and 95% confidence interval (95% CI). Respiratory parameters that were statistically significant after multivariate Cox analysis, were elected for formation of a nomogram which proportionally converted each multivariate regression coefficient to a 0-100 point scale.

Model formation

The RAS model was established with scoring points allotted from 1 to 10 after the above multivariate regression was utilized to determine the relative points for each significant respiratory parameter to reflect their impact on the progression of disease. The prognostic parameters were classified based on normal range and values for RR and resting O2 saturation while for A-a gradient an elevated value with respect to normal for age was considered noteworthy [11], as outlined in Table 1.

**Table 1:** RAS calculator. RAS, respiratory assessment scoring; b/m, breaths/minute; A-a Gradient, alveolar-arterial oxygen gradient.

	Points
Respiratory rate (b/m)	
≤20	1
21-25	2
26-30	3
>30	4
Resting oxygen saturation (%)	
≥95	1
90-94	2
<90	3
A-a Gradient	
Normal	1
Elevated	3

In addition, receiver operating characteristic (ROC) curves were used to evaluate the performance of the RAS prognostic model along with computation of area under the ROC (AUROC). Optimal cutoff values for progression and mortality of disease were also calculated and assessed by sensitivity, specificity, predictive values, and likelihood ratios.

## Results

Clinical characteristics

Overall, 252 COVID-19 patients were entered in the study, median age 59 (IQR: 50-70), among which the majority were males (86.5%). Out of these 252 cases, 180 (71.4%) had comorbidities which mainly included hypertension (32.8%), diabetes mellitus (30.6%), and cardiovascular disease (23.3%). History of contact with confirmed COVID-19 cases was noted in 69 (27.4%) patients. Among the reported symptoms cough (78.2%) was the commonest, followed by fever (75.8%), shortness of breath (66.3%), body aches (20.2%), and diarrhea (7.9%). Progression of illness was noted in 60% of patients who originally presented with shortness of breath (p <0.001). HRCT chest showed classic findings for COVID-19 in 181 (71.8%) cases, while 23 (9.1%) had atypical and 24 (9.5%) had mixed findings. In 134 (53.2%) patients, lung involvement was over 50%, of which 70.9% worsened and 28.4% expired (p <0.001). Based on established categories, at admission 115 (45.6%) patients were classified to have severe disease, 99 (39.3%) moderate, 22 (8.7%) critical disease, and only 16 (6.3%) to mild group with comorbidities. Overall, progression of the disease appeared in 124 (49.2%) cases who consisted mainly of patients with severe disease category (63%), followed by patients with critical disease (17.7%), moderate disease (14.5%), and a few with mild disease with comorbidities (4.8%). Some 100% of patients in our study with critical initial disease category eventually died. Clinical attributes of patients were studied among stable, worsened and mortality associated groups, values of which are revealed in Table 2.

**Table 2:** Characteristics of enrolled patients and their correlation with progression of disease and mortality. Skewedly distributed continuous variables are expressed as medians (interquartile range, IQR) and compared with Mann-Whitney U test; categorical variables are presented as n (%) and compared by the chi-square test. COPD, chronic obstructive pulmonary disease; CLD, chronic liver disease; CRF, chronic renal failure; SOB, shortness of breath; LDH, lactate dehydrogenase; b/m, breaths per minute; HRCT, high resolution computed tomography; O_2_, oxygen; A-a gradient, alveolar to arterial oxygen gradient.

	Overall (n = 252)	Stable (n = 128)	Worsened (n = 124)	p-Value	Death (n = 49)	p-Value
Age, years	59 (50-70)	56 (48-68)	62 (53-72)	0.005	70 (58-75)	0.005
Comorbidity	180 (71.4)	92 (71.9)	88 (71)	0.873	43 (87.8)	0.005
Diabetes	55 (21.8)	28 (21.9)	27 (21.8)	0.985	9 (18.4)	0.514
Hypertension	59 (23.4)	33 (25.8)	26 (21)	0.367	11 (22.4)	0.859
Cardiovascular disease	42 (16.7)	23 (18)	19 (15.3)	0.573	7 (14.3)	0.618
Asthma/COPD	9 (3.6)	5 (3.9)	4 (3.2)	0.771	4 (8.2)	0.054
CLD	3 (1.2)	0 (0)	3 (2.4)	0.077	2 (4)	0.038
CRF	2 (0.8)	1 (0.8)	1 (0.8)	0.982	1 (2)	0.273
Fever	191 (75.8)	87 (68)	104 (83.9)	0.003	42 (85.7)	0.071
Cough	197 (78.2)	96 (75)	101 (81.5)	0.215	38 (77.6)	0.906
Dyspnea	167 (66.3)	66 (51.6)	101 (81.5)	<0.001	43 (87.8)	<0.001
Lymphocyte count, x10^9^/L	1.2 (0.7-1.5)	1.3 (0.9-1.6)	0.8 (0.7-1.3)	<0.001	0.8 (0.7-1.1)	<0.001
LDH, U/L	440 (289-789)	358 (231-554)	592 (365-928)	<0.001	678 (440-1009)	<0.001
Respiratory rate, b/m	20 (16-31)	17 (13-21)	30 (21-33)	<0.001	33 (31-36)	<0.001
Resting O_2_ saturation, %	92 (86-96)	94 (92-97)	88 (82-92)	<0.001	81 (79-85)	<0.001
A-a gradient, mmHg	39 (12-76)	17 (9-39)	69 (39-98)	<0.001	80 (48-107)	<0.001
10 feet O_2 _desaturation test, %	6 (2-11)	3 (1-7)	9 (4-14)	<0.001	15 (9-16)	<0.001
Initial disease category				<0.001		<0.001
Mild with comorbidities	16 (6.3)	10 (7.8)	6 (4.8)		2 (4)	
Moderate	99 (39.3)	81 (63.6)	18 (14.5)		4 (8.2)	
Severe	115 (45.6)	37 (29)	78 (62.9)		21 (42.9)	
Critical	22 (8.7)	0 (0)	22 (17.7)		22 (44.9)	
HRCT chest findings				0.074		0.331
Normal	24 (9.5)	18 (14)	6 (4.8)		2 (4)	
Typical	181 (71.8)	86 (67.2)	95 (76.6)		35 (71.4)	
Atypical	23 (9.1)	13 (10.2)	10 (8)		5 (10.2)	
Mixed	24 (9.5)	11 (8.6)	13 (10.5)		7 (14.3)	
Lung involvement				<0.001		<0.001
Normal	21 (8.3)	17 (13.3)	4 (3.2)		1 (2)	
<50%	97 (38.5)	72 (56.3)	25 (20.2)		10 (20.4)	
>50%	134 (53.2)	39 (30.5)	95 (76.6)		38 (77.6)	
Age				<0.001		<0.001
≤60 years	130 (51.6)	80 (62.5)	50 (40.3)		11 (22.4)	
>60 years	122 (48.4)	48(37.5)	74 (59.7)		38 (77.6)	
Lymphocyte count				<0.001		<0.001
>1.0 x 10^9^/L	143 (56.7)	91 (71.1)	52 (41.9)		16 (32.7)	
≤1.0 x 10^9^/L	109 (43.3)	37 (28.9)	72 (58)		33 (67.3)	
LDH				<0.001		0.001
≤250 U/L	49 (19.4)	38 (29.7)	11 (8.9)		4 (8.2)	
250-500 U/L	106 (42.1)	57 (44.5)	49 (39.5)		15 (30.6)	
>500 U/L	97 (38.5)	33 (25.8)	64 (51.6)		30 (61.2)	
Respiratory rate				<0.001		<0.001
≤20 b/m	126 (50)	95 (74.2)	31 (25)		3 (6.1)	
21-25 b/m	39 (15.5)	21 (16.4)	18 (14.5)		3 (6.1)	
26-30 b/m	21 (8.3)	5 (3.9)	16 (12.9)		5 (10.2)	
>30 b/m	66 (26.2)	7 (5.5)	59 (47.6)		38 (77.6)	
Resting O_2_ saturation				<0.001		<0.001
≥95%	77 (30.6)	63 (49.2)	14 (11.3)		1 (2)	
90-94%	80 (31.7)	48 (37.5)	32 (25.8)		7 (14.3)	
<90%	95 (37.7)	17 (13.3)	78 (62.9)		41 (83.7)	
A-a gradient				<0.001		<0.001
Normal	80 (31.7)	67 (52.3)	13 (10.5)		0	
Elevated	172 (68.3)	61 (47.7)	111 (89.5)		49 (100)	
10 feet O_2 _desaturation test				<0.001		<0.001
<3%	79 (31.3)	62 (48.4)	17 (13.7)		2 (4)	
≥3%	173 (68.7)	66 (51.6)	107 (86.3)		47 (96)	
Days in hospital	10 (7-12)	8 (6-11)	11 (9-13)	<0.001	10 (8-12)	0.609

Regression analysis and construction of nomogram

**Figure 1:**
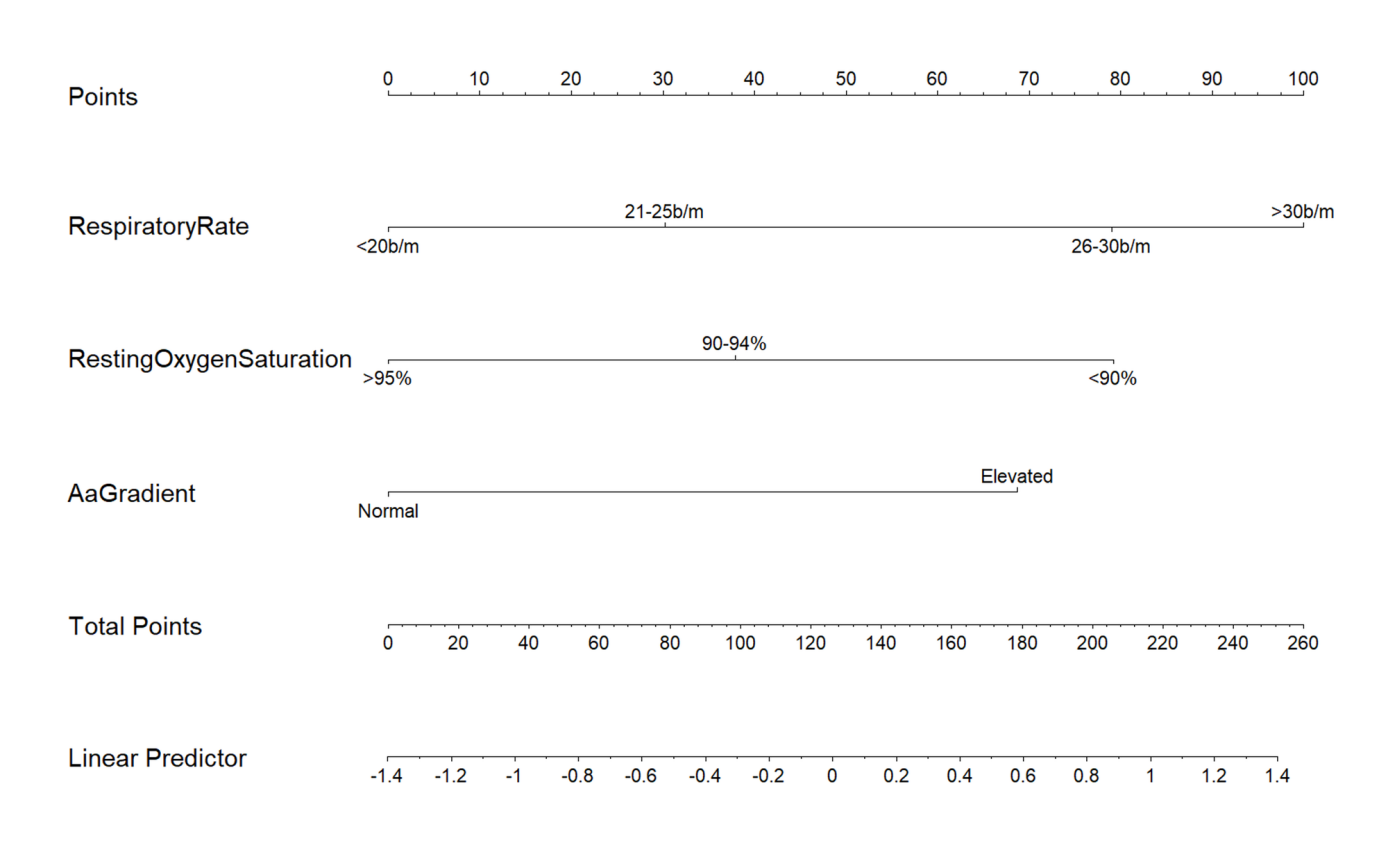
Formulated nomogram for prediction of worsening disease. The value of each Respiratory Assessment Scoring (RAS) model variable is given a certain score on a point scale from 0 to 100; to utilize the nomogram, value of each variable is determined on corresponding axis, a line is drawn to the point axis, points from all the variables are added, and total points are projected to the linear predictor. b/m, breaths per minute.

Univariate and multivariate Cox regression analysis showed that 10 feet O2 desaturation test (HR: 0.99, 95% CI: 0.95-1.04, p=0.706) is not a powerful predictor of progression of disease; however, it substantiates that increasing respiratory rate (HR: 1.06, 95% CI: 1.03-1.09, p<0.001), decreasing resting oxygen saturation (HR: 0.95, 95% CI: 0.91-0.99, p=0.019) and greater than normal A-a gradient (HR: 1.00, 95% CI: 1.0-1.15, p=0.022) are independent high-risk factors associated with progression of illness. Based on these factors, a predictive nomogram (Figure 1) was formulated, and each variable was categorized to express its weight of impact on progression of the illness.

Respiratory rate was divided into four levels; less than and equal to 20 b/m, 21 to 25 b/m (HR: 1.40, 95% CI: 0.76-2.57), 26 to 30 b/m (HR: 2.40, 95%CI: 1.24-4.64) and more than 30 b/m (HR: 3.03, 95%CI: 1.77-5.19). Resting O2 saturation was grouped as more than and equal to 95%, 90%-94% (HR: 1.52, 95% CI: 0.76-3.06) and less than 90% (HR: 2.41, 95%CI: 1.15-5.06). For A-a gradient a value higher than normal for age was regarded as a cutoff (HR: 2.14, 95% CI: 1.04-4.39), as illustrated in Table 3.

**Table 3:** Univariate and multivariate Cox proportional hazards regression analysis of progression of illness with CALL and RAS Score parameters. HRs were calculated comparing without comorbidity versus with comorbidity, age ≤60 years versus >60 years, lymphocyte count >1.0 x 10/L versus ≤1.0 x 10/L, LDH ≤250 U/L versus LDH 250-500 U/L or >500 U/L, respiratory rate ≤20 b/m versus 21-25 b/m or 26-30 b/m or >30 b/m, resting oxygen saturation ≥95% versus 90%-94% or <90%, normal A-a gradient versus elevated A-a gradient, 10 feet O_2 _desaturation <3% versus ≥3%. HR, hazard ratio; CALL, comorbidity, age, lymphocyte and LDH; RAS, respiratory assessment scoring; LDH, lactate dehydrogenase; b/m, breaths per minute; A-a gradient, alveolar arterial oxygen gradient; O_2_, oxygen.

	Univariate Cox analysis HR (95% CI) p-Value		Multivariate Cox analysis HR (95% CI) p-Value	
Comorbidity				
Without	1	_	1	_
With	0.97 (0.66-1.43)	0.876	0.89 (0.59-1.33)	0.564
Age (Years)				
≤60	1	_	1	_
>60	1.87 (1.30-2.68)	<0.001	1.50 (1.02-2.22)	0.041
Lymphocyte (x10^9^/L)				
>1.0	1	_	1	_
≤1.0	2.30 (1.61-3.29)	<0.001	1.76 (1.20-2.57)	0.003
LDH (U/L)				
≤250	1	_	1	_
250-500	2.43 (1.26-4.68)	0.008	2.17 (1.13-4.20)	0.020
>500	4.09 (2.15-7.75)	<0.001	3.33 (1.74-6.37)	<0.001
Respiratory rate (b/m)				
≤20	1	_	1	_
21-25	2.21 (1.24-3.95)	0.007	1.40 (0.76-2.57)	0.281
26-30	4.85 (2.65-8.90)	<0.001	2.40 (1.24-4.64)	0.009
>30	6.66 (4.28-10.38)	<0.001	3.03 (1.77-5.19)	<0.001
Resting oxygen saturation (%)				
≥95	1	_	1	_
90-94	2.57 (1.37-4.82)	0.003	1.52 (0.76-3.06)	0.239
<90	7.83 (4.41-13.89)	<0.001	2.41 (1.15-5.06)	0.020
A-a Gradient				
Normal	1	_	1	_
Elevated	5.71 (3.21-10.17)	<0.001	2.14 (1.04-4.39)	0.038
10 feet O_2_ desaturation test (%)				
<3	1	_	1	_
≥3	3.89 (2.33-6.49)	<0.001	1.44 (0.78-2.62)	0.227

Cox regression test on variables of the CALL score indicated that age older than 60 years (HR: 1.5, 95% CI: 1.02-2.22, p=0.041), lymphocyte count of less than 1.0 x 109/L (HR: 1.76, 95% CI: 1.20-2.57, p=0.003), LDH of 250-500 U/L (HR: 2.17, 95% CI: 1.13-4.20, p=0.020), and LDH of more than 500 U/L (HR: 3.33, 95% CI: 1.74-6.37, p<0.001) were also high-risk elements linked with disease progression. On the other hand, presence of comorbidity revealed a p=0.565 on multivariate regression for progression of illness.

Model assessment

**Figure 2:**
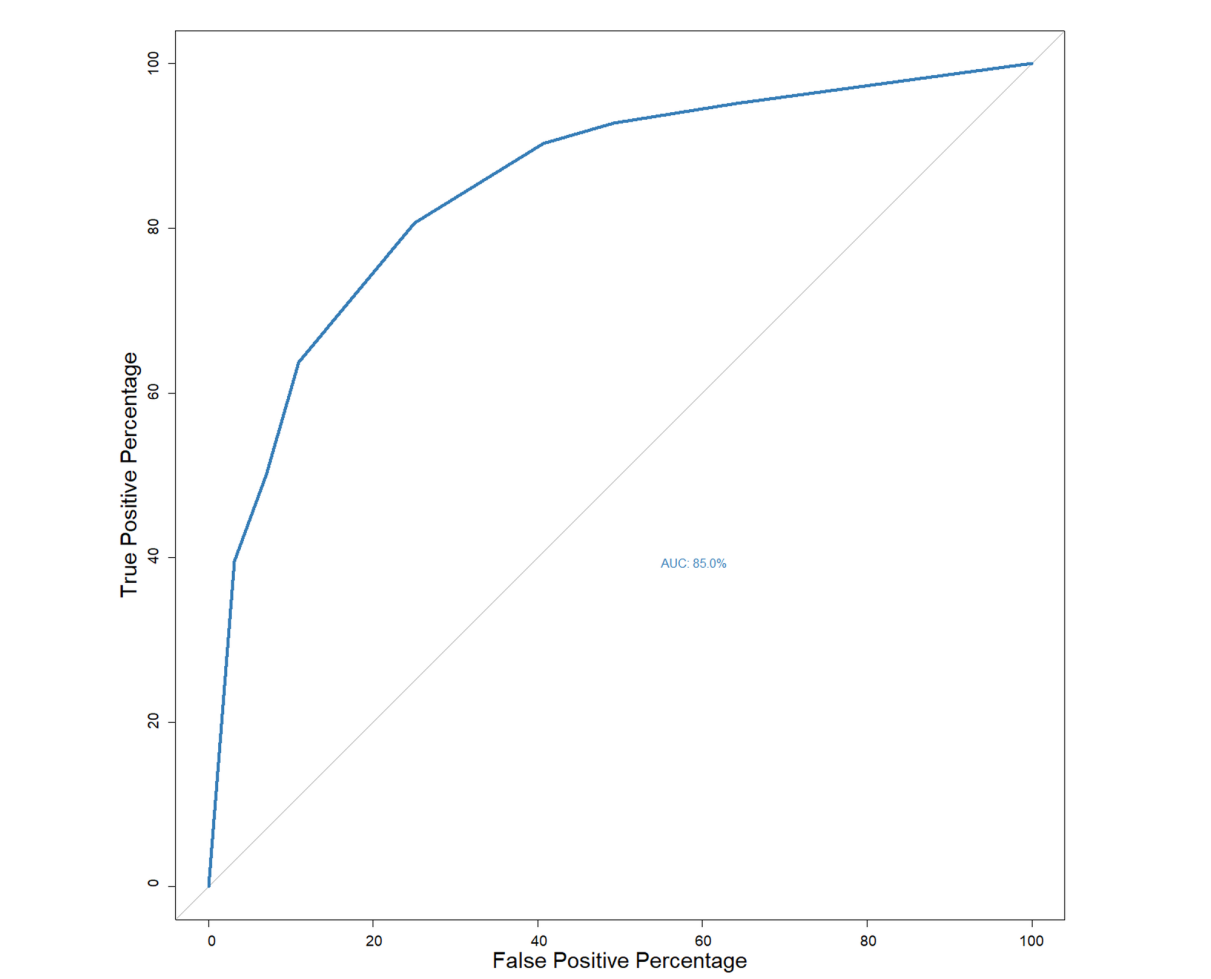
ROC curve correlating RAS Score with progression of illness. ROC, receiver operating characteristics; RAS, respiratory assessment scoring; AUC, area under the curve.

Performance of the RAS model was checked using ROC curve where area under the ROC (AUROC) was 85% (95%CI: 80%-89%) (Figure 2). A cutoff value of seven points was accepted for disease progression whose positive-predictive value (95% CI) was 76% (68%-83%) and negative-predictive value (95%CI) was 80% (73%-87%) for prediction (Table 4). Using a cutoff value of nine points for mortality associated with the disease, the positive predictive value (95%CI) was 55% (43%-67%) and the negative predictive value (95%CI) was 95% (91%-98%) (Table 5).

**Table 4:** Accuracy of RAS Model for estimating risk of progression of disease. RAS, respiratory assessment scoring; AUROC, area under the receiver operating characteristic curve; CI, confidence interval.

Variable	Enrolled patients (n=252)
AUROC, % (95% CI)	85 (80-89)
Cutoff value	7
Sensitivity, % (95% CI)	81 (74-88)
Specificity, % (95% CI)	75 (68-83)
Positive predictive value, % (95% CI)	76 (68-83)
Negative predictive value, % (95% CI)	80 (73-87)
Positive likelihood ratio (95% CI)	3.23 (2.36-4.41)
Negative likelihood ratio (95% CI)	0.26 (0.18-0.38)

**Table 5:** Accuracy of RAS Model for estimating mortality. RAS, respiratory assessment scoring; AUROC, area under the receiver operating characteristic curve; CI, confidence interval.

Variable	Enrolled patients (n = 252)
AUROC, % (95% CI)	90 (86-94)
Cutoff value	9
Sensitivity, % (95% CI)	80 (68-91)
Specificity, % (95% CI)	84 (79-89)
Positive predictive value, % (95% CI)	55 (43-67)
Negative predictive value, % (95% CI)	95 (91-98)
Positive likelihood ratio (95% CI)	5.05 (3.57-7.15)
Negative likelihood ratio (95% CI)	0.24 (0.14-0.42)

Additionally, the RAS score was divided into three groups based on their chances to progression; scores of 3-6 points (class A) had less than 15% probability of progression, 6-8 points were considered intermediate risk (class B) because of 15%-50% possibility of progression and high risk (class C) was appointed to set of 9-10 points with over 70% probability of worsening condition.

On day zero, 16.3% patients of the study fell in the CALL score category A, 38.1% in B and 45.6% in C, among which 36.6%, 31.2%, and 68.7% lead to progression of disease in A, B, and C categories, respectively. Like-wise, on day zero 88 (34.9%), 93 (36.9%), and 71 (28.2%) patients belonged to category A, B, and C on RAS score, respectively. Progression was observed in an increased number of patients in category C (87.3%) followed by category B (53.8%) and category A patients (13.6%). Similarly, mortality figures were nonexistent in the RAS A group and considerably lower in RAS B (10.8%) than the RAS C category (54.9%).

## Discussion

COVID-19 is a novel infectious disease that has spread throughout the world, resulting in over one million deaths in the course of a few months. Timely identification of patients who are at risk of progression to severe disease and mortality, can lead to better management and favorable outcomes. The purpose of this study was to appraise the potency of the CALL prognostic model [6] and to build an alternative scoring system based on the measurable respiratory parameters.

Key findings

The 252 COVID-19 patients in this study consisted of an increased amount of people of old age and with presence of comorbidities. Both of these groups of people were noted to have significant association with mortality. People who originally presented with shortness of breath and increased pulmonary infiltrates on radiographs were more prone to severe disease and mortality. Decreased lymphocyte count, raised LDH, increased RR, lower oxygen saturation at rest, elevated A-a gradient, oxygen desaturation on minimal exertion, and severe initial disease category also revealed a significant correlation with disease progression and death. In this study population, the CALL score proved to be a reliable prognosticator of worsening illness and associated fatality. However, multivariate regression analysis on variables of CALL score did not approve the presence of comorbidity to be a significant high-risk predictor of progression of the illness.

The Respiratory Assessment Scoring (RAS) system was developed based on three measurable high-risk respiratory parameters, namely; RR, resting oxygen saturation, and A-a gradient, which could identify a group of patients with either increased likelihood of stability, or worsening of disease and death. All three variables of the RAS model showed significant relation with the progression of disease on Cox regression studies.

Comparison with existing literature

The median age (IQR) for patients who died in this study was 70 (58-75) years, which is similar to many studies where older age is linked with rising mortality as evident in the narrative review by Stawicki et al. [7]. In conjunction with the results, lymphocyte count has been a remarkably well-validated variable employed in various studies, the lower count being an indicator of worse outcome [2,12-14]. Elevated LDH values have likewise been associated with a rise in odds of mortality, identical to findings in this study [15-17]. The presence of lung infiltrates was noticed to have a significant correlation with progression and death, which justifies its value in already established models [18-19].

In one study, the CALL score has turned out to have a good prognostic index for in-hospital mortality but not for disease progression [20]. However, a significant amount of patients with higher CALL scores in this study population developed progression of disease, as Ji et al. said that CALL score with a cut off value of nine points showed positive and negative predictive values of 78.3% and 11.9% respectively [6]. It was observed that the three variables of CALL score; age more than 60 years, lymphocyte count of less than 1.0 x 109/L and increased LDH, were reliable predictors of worsening disease condition as put forth by Ji et al. [6]. In contrast, the presence of comorbidity alone was not found to be a reliable independent risk factor of disease progression in this sample of 252 patients, which falls in disagreement with many studies that previous comorbid conditions in coronavirus patients are correlated with worsening disease and mortality [2-4, 7, 21]. A few predictive models have also failed to establish comorbidity as a prognosticator of disease severity, suggesting a demand to properly establish and validate the effect of previous disease conditions in COVID-19 patients [22-23].

While several predictive models have been produced for COVID-19, only one scoring model has been found to integrate simple and clinically useful parameters such as increased respiratory rate and reduced oxygen saturation to assess disease progression, however, this said Brescia-COVID Respiratory Severity Scale (BCRSS) was a rapidly established score that has not yet been validated [19]. RAS model specifications, i.e. respiratory rate and oxygen saturation has been used as a response to noninvasive ventilation in COVID-19 patients [19, 24]. These along with and A-a gradient have also been already validated to monitor respiratory failure in intensive care setups [25].

Strengths and limitations

The RAS model was used as an investigational score in this study. It has a high negative predictive value for mortality and fair negative and positive predictive values for disease progression. It can facilitate better-informed discussions between clinicians and patients’ families about the anticipated clinical trajectory, allowing accurate and timely advance-care planning.

This study was a pilot study for the RAS carried out at a single center with a number of limitations. Firstly, the sample size was small and secondly, a prospective study is also warranted to test the validity and efficacy of this model. Also, the 10 feet desaturation test that was devised and used, is not an otherwise validated tool for respiratory assessment. However, it has been found that in a substantial group of patients with COVID-19, hypoxia becomes apparent only on exertion [26]. Since some subjects in this sample population were already severely hypoxemic on rest, the validity of this test is also questionable. Nevertheless, due to the significant association of exertional desaturation observed with disease progression and fatality, it is paramount to provide a safe and effective minimal exertional desaturation test for coronavirus patients, also emphasized by Greenhalgh et al. [9].

## Conclusions

The CALL and RAS scores achieved significant predictive power of disease progression and mortality in this study. Nonetheless, more prospective studies are required for validation of these scores, in order to justify their use in the setting of this devastating disease. Moreover, the importance and role of comorbidities and minimal exercise oxygen desaturation tests need to be further investigated to explain and validate their role in COVID-19 patients. The spectrum and implications of the novel coronavirus disease ought to be explored in detail and research on larger sample populations using multi-center studies is required to evince authentic knowledge.
